# Perrault syndrome with neurological features in a compound heterozygote for two *TWNK* mutations: overlap of *TWNK*-related recessive disorders

**DOI:** 10.1186/s12967-019-2041-x

**Published:** 2019-08-28

**Authors:** María Domínguez-Ruiz, Alberto García-Martínez, Marc Corral-Juan, Ángel I. Pérez-Álvarez, Ana M. Plasencia, Manuela Villamar, Miguel A. Moreno-Pelayo, Antoni Matilla-Dueñas, Manuel Menéndez-González, Ignacio del Castillo

**Affiliations:** 1 Servicio de Genética, Hospital Universitario Ramón y Cajal, IRYCIS, Carretera de Colmenar km 9.100, 28034 Madrid, Spain; 2 Centro de Investigación Biomédica en Red de Enfermedades Raras (CIBERER), Madrid, Spain; 3 Department of Neurology, Servicio de Neurología, Hospital Universitario Central de Asturias, Avenida Roma sn, 33011 Oviedo, Spain; 4 Functional and Translational Neurogenetics Unit, Department of Neuroscience, Health Sciences Research Institute Germans Trias i Pujol (IGTP), Universitat Autónoma de Barcelona, Can Ruti Campus, Badalona, Barcelona Spain; 5 Servicio de Pediatría, Hospital Universitario Central de Asturias, Oviedo, Spain

**Keywords:** Perrault syndrome, Hearing impairment, Polyneuropathy, Ataxia, Premature ovarian failure, *TWNK*, Mitochondrial DNA helicase

## Abstract

**Background:**

Perrault syndrome is a rare autosomal recessive disorder that is characterized by the association of sensorineural hearing impairment and ovarian dysgenesis in females, whereas males have only hearing impairment. In some cases, patients present with a diversity of neurological signs. To date, mutations in six genes are known to cause Perrault syndrome, but they do not explain all clinically-diagnosed cases. In addition, the number of reported cases and the spectra of mutations are still small to establish conclusive genotype–phenotype correlations.

**Methods:**

Affected siblings from family SH19, who presented with features that were suggestive of Perrault syndrome, were subjected to audiological, neurological and gynecological examination. The genetic study included genotyping and haplotype analysis for microsatellite markers close to the genes involved in Perrault syndrome, whole-exome sequencing, and Sanger sequencing of the coding region of the *TWNK* gene.

**Results:**

Three siblings from family SH19 shared similar clinical features: childhood-onset bilateral sensorineural hearing impairment, which progressed to profound deafness in the second decade of life; neurological signs (spinocerebellar ataxia, polyneuropathy), with onset in the fourth decade of life in the two females and at age 20 years in the male; gonadal dysfunction with early cessation of menses in the two females. The genetic study revealed two compound heterozygous pathogenic mutations in the *TWNK* gene in the three affected subjects: c.85C>T (p.Arg29*), previously reported in a case of hepatocerebral syndrome; and a novel missense mutation, c.1886C>T (p.Ser629Phe). Mutations segregated in the family according to an autosomal recessive inheritance pattern.

**Conclusions:**

Our results further illustrate the utility of genetic testing as a tool to confirm a tentative clinical diagnosis of Perrault syndrome. Studies on genotype–phenotype correlation from the hitherto reported cases indicate that patients with Perrault syndrome caused by *TWNK* mutations will manifest neurological signs in adulthood. Molecular and clinical characterization of novel cases of recessive disorders caused by *TWNK* mutations is strongly needed to get further insight into the genotype–phenotype correlations of a phenotypic continuum encompassing Perrault syndrome, infantile-onset spinocerebellar ataxia, and hepatocerebral syndrome.

## Background

Perrault syndrome (PRLTS) is an autosomal recessive genetic disorder that is characterized by the association of sensorineural hearing impairment and ovarian dysgenesis in females [1, 2]. Affected males may have only an apparently non-syndromic hearing impairment. In some cases, males and females present with neurological signs, including progressive sensory and motor peripheral neuropathy, cerebellar ataxia, and mild intellectual disability [2, 3]. Accordingly, PRLTS is classified clinically into two types, with or without neurologic manifestations (types II and I, respectively). PRLTS is a rare disease, although no accurate data on its prevalence are available. Given that hearing impairment may be the only clinical sign in males, underascertainment is likely [2].

PRLTS is genetically heterogeneous. To date, mutations in six genes are known to cause different genetic types of the syndrome (Table 1).

**Table 1:** Genes known to be involved in Perrault syndrome

Genetic type	MIM entry	Gene	Gene MIM entry	Location
PRLTS1	233400	*HSD17B4*	601860	5q23.1
PRLTS2	614926	*HARS2*	600783	5q31.3
PRLTS3	614129	*CLPP*	601119	19p13.3
PRLTS4	615300	*LARS2*	604544	3p21.31
PRLTS5	616138	*TWNK*	606075	10q24.31
PRLTS6	617565	*ERAL1*	607435	17q11.2

*HSD17B4*, the first gene whose involvement in PRLTS was reported [4], encodes 17β-hydroxysteroid dehydrogenase type 4, a peroxisomal multifunctional enzyme involved in the fatty acid β-oxidation pathway and steroid metabolism. The other five genes code for proteins that play roles in mitochondrial function. *HARS2* [5] and *LARS2* [6] code for mitochondrial histidyl-tRNA synthetase 2 and leucyl-tRNA synthetase 2, respectively. *CLPP* [7] encodes an endopeptidase subunit of a mitochondrial ATP-dependent proteolytic complex. *TWNK* [8] encodes Twinkle, a DNA helicase that is essential for the replication of the mitochondrial genome. Finally, *ERAL1* [9] codes for a GTPase that acts as a chaperone for the 12S mitochondrial rRNA, and so it contributes to the proper assembly of the 28S small mitochondrial ribosomal subunit. After screening for mutations in these six genes, molecular diagnosis is not reached in approximately 60% of individuals with PRLTS [2], which indicates that its genetic bases are still far from being completely understood.

The number of cases with mutations in each of the six known genes is still small to establish definite genotype–phenotype correlations. So far, all patients with PRLTS due to mutations in *HSD17B4* and *TWNK* present with neurological manifestations (clinical type II) [4, 8, 10–14]. Mutations in *CLPP* or *LARS2* may result in clinical types I or II [6, 7, 12, 14–20]. Finally, no neurological manifestations have been reported to date in patients with mutations in *HARS2* or *ERAL1* [5, 9, 14], but these are the genes with fewer known cases. Molecular characterization of novel cases is needed to progress in finding correlations. Here we report a Spanish familial case of Perrault syndrome with neurological manifestations and show it is caused by two compound heterozygous mutations in the *TWNK* gene, one of which is novel whereas the other was previously reported in a case of hepatocerebral syndrome. Implications of these findings in the genotype–phenotype correlations of *TWNK* mutations are discussed.

## Methods

Affected subjects from family SH19 were clinically examined by the Departments of Pediatrics and Neurology of Hospital Universitario Central de Asturias. The genetic study was performed in parallel by the Service of Genetics of the Hospital Universitario Ramón y Cajal, and by the Neurogenetics Unit of the Research Institute Germans Trias i Pujol (IGTP) in Badalona (Barcelona).

DNA was extracted from peripheral blood samples from all members of each family by standard methods. Microsatellite markers flanking the genes involved in PRLTS were chosen from the literature [21] and electronic databases [22] (Table 2), and were amplified using fluorescently-labeled primers and PCR conditions as previously reported. Amplified alleles were resolved by capillary electrophoresis in an ABI Prism 3100 Avant Genetic Analyzer (Applied Biosystems).

**Table 2:** Microsatellite markers that map close to the genes involved in PRLTS

Gene	Microsatellite markers
*HSD17B4*	D5S404, D5S494, D5S471, D5S503
*HARS2*	D5S2116, D5S658, D5S2010
*CLPP*	D19S1034, D19S427, D19S406, D19S873
*LARS2*	D3S3624, D3S3582, D3S1581
*TWNK*	D10S192, D10S1265, D10S1697
*ERAL1*	D17S1878, D17S925, D17S1873

Whole-exome sequencing (WES) was performed on genomic DNA obtained from peripheral blood. Exome capture was performed using SureSelect All Human Enrichment Target Exon (Agilent Technologies) for 71 Mb according to the manufacturer’s protocol. Paired-end, 101-nt long reads were generated on a HiSeq 2000 platform (Illumina Inc.). Read alignment was performed using BWA (Burrows-Wheeler Aligner) [23]. Variant calling was performed using a combination of two different algorithms: VarScan [24] and GATK [25]. Identified variants were annotated using the Ensembl database [26]. Variants with a minor allele frequency ≥ 1% in genetic databases including the Exome Aggregation Consortium, 1000-Genomes Project and dbSNP, were excluded from further analysis. The genetic heterogeneity model proposed by Ng et al. [27] was applied in order to identify potential candidate genes. Candidate variants were assessed for their computationally predicted pathogenicity by SIFT, PolyPhen-2, and MutationTaster [28–30], and prioritized in accordance with the clinical characteristics shared by affected siblings. Segregation of candidate variants was confirmed by Sanger sequencing.

Primer sequences for PCR amplification of all exons and exon–intron boundaries of the *TWNK* gene are listed in Table 3.

**Table 3:** Primers for PCR amplification of *TWNK* exons and exon–intron boundaries

Exon	Sequence of primers	Annealing temperature (°C)	Amplicon size (bp)
1 (part 1)	F: 5′-CTAAGCAGCGAGGAGAGGGGG-3′ R: 5′-TCGCCACGTCCTTCTACAAT-3′	63	543
1 (part 2)	F: 5′-GAGGACGACGAGGAGATGCTGTG-3′ R: 5′-CCCATGCCCCCGCAAATAC-3′	63	571
1 (part 3)	F: 5′-TGCGTGGGGAGTGGATGG-3′ R: 5′-GCTGGGTCGGGGAATAGTGGT-3′	63	677
1 (part 4)	F: 5′-GTGTGCGATATCTGCGACCTG-3′ R: 5′-CATTGTCATGGCCCAAACCC-3′	63	746
2	F: 5′-TTTGGGCCATGACAATGTT-3′ R: 5′-GGAGATGGGGGAGTTCCTAC-3′	60	467
3	F: 5′-CTTCTGCCTGGGGTGGTC-3′ R: 5′-TCCATACATCCTGCTGCCATAC-3′	60	348
4	F: 5′-GTATGGCAGCAGGATGTATGGA-3′ R: 5′-TGTTCGGATGGACAGTCAAGA-3′	60	413
5	F: 5′-CCTCTCCCCATTCTTATCACT-3′ R: 5′-AGCTCAGGACCACAGGATAG-3′	60	518

*F* forward, *R* reverse

Mutation nomenclature is annotated based on cDNA sequence (GenBank accession number NM_021830.4) and according to the current Human Genome Variation Society rules as implemented by the Mutalyzer 2.0.3 program (Leiden University Medical Center, Leiden, The Netherlands).

## Results

**Fig. 1:**
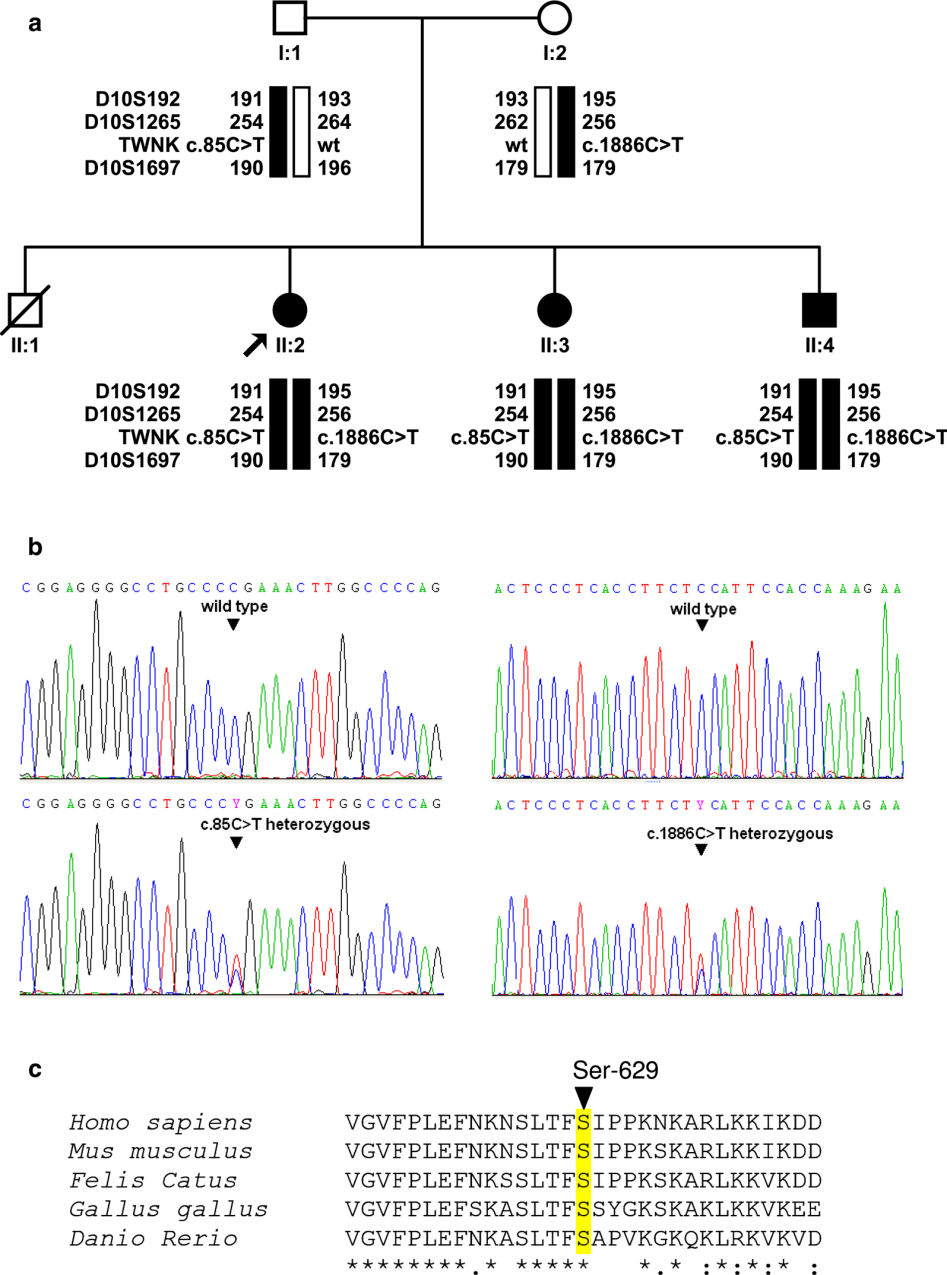
Genetic study of family SH19. **a** Pedigree, haplotype analysis for microsatellite markers and segregation of mutations in the *TWNK* gene. **b** Electropherograms of the two heterozygous mutations that were found in *TWNK* in the family. **c** Alignment of twinkle orthologous sequences from five model organisms: human (accession number NP_068602.2), mouse (NP_722491.2), cat (XP_003994377.1), chick (NP_001026515.1), and zebrafish (NP_001264527.1). Only stretches aligning to human twinkle residues 614–644 are shown. Asterisks indicate identical residues across all sequences; colons, conserved positions (residues of strongly similar properties); periods, semi-conserved positions (residues of weakly similar properties). The location of the residue affected by the p.Ser629Phe mutation is indicated by an arrowhead

Family SH19 included four siblings who were born from non-consanguineous parents (Fig. 1a). The eldest brother (subject II:1) died from purulent meningitis at age 21 months. The remaining three siblings (two sisters, one brother) shared similar clinical features, including spinocerebellar ataxia, polyneuropathy, sensorineural hearing impairment, and gonadal dysfunction in females, as detailed below.

Subject II:2. At age 5 years, she developed a progressive hearing loss leading to profound bilateral deafness by age 15. At age 20, her menses ceased, but the subsequent gynaecological study did not result in an etiologic diagnosis. At age 35 she started to experience difficulties with walking, but she did not visit a neurologist until age 43. On examination we confirmed a profound hearing impairment, and found fast horizontal and vertical nystagmus. Standing and gait were unstable, and she was not able to perform tandem gait. Romberg test was positive. She had cavus feet and universal areflexia. Touch and arthrokinetic sensitivity were diminished in lower limbs with a distal gradient, whereas the algesic and vibratory sensitivities were preserved. Over time, gait became impaired and showed ataxic features. There was also an impairment of arthrokinetic sensitivity in upper limbs. Later on we found global hypotony and mild atrophy in the four limbs, as well as dysarthria. Neuroimaging studies found a thinning of the spinal cord, cerebellar atrophy and signal changes in the cerebellar white matter.

Subject II:3. At age 3 years, she developed a progressive sensorineural hearing loss leading to profound bilateral deafness by age 13. At age 32, she suffered a traffic accident that resulted in a traumatic head injury, with no sequels. Soon after, her menses ceased. At age 43 she visited the neurologist because of difficulties with walking. We confirmed the profound hearing impairment, and found dysarthria, global hypotony, universal areflexia and loss of arthrokinetic sensitivity in the feet, which were cavus. There was dysmetria in cerebellar manoeuvres with lower limbs, and she was unable to perform tandem gait. Neurophysiologic studies found conduction defects bilaterally in the auditory pathways, in the right visual pathway, and in the somesthetic pathway at the posterior spinal cords for afferents from both lower and upper limbs. Electroneurography showed symmetric demyelinating sensitive polyneuropathy. Brain MRI showed global cerebellar atrophy and signal changes in the cerebellar white matter, particularly in the middle cerebellar peduncle. The patient has also been diagnosed with autoimmune hypothyroidism secondary to Hashimoto thyroiditis, vitiligo and increase of liver enzymes of unknown cause.

**Fig. 2:**
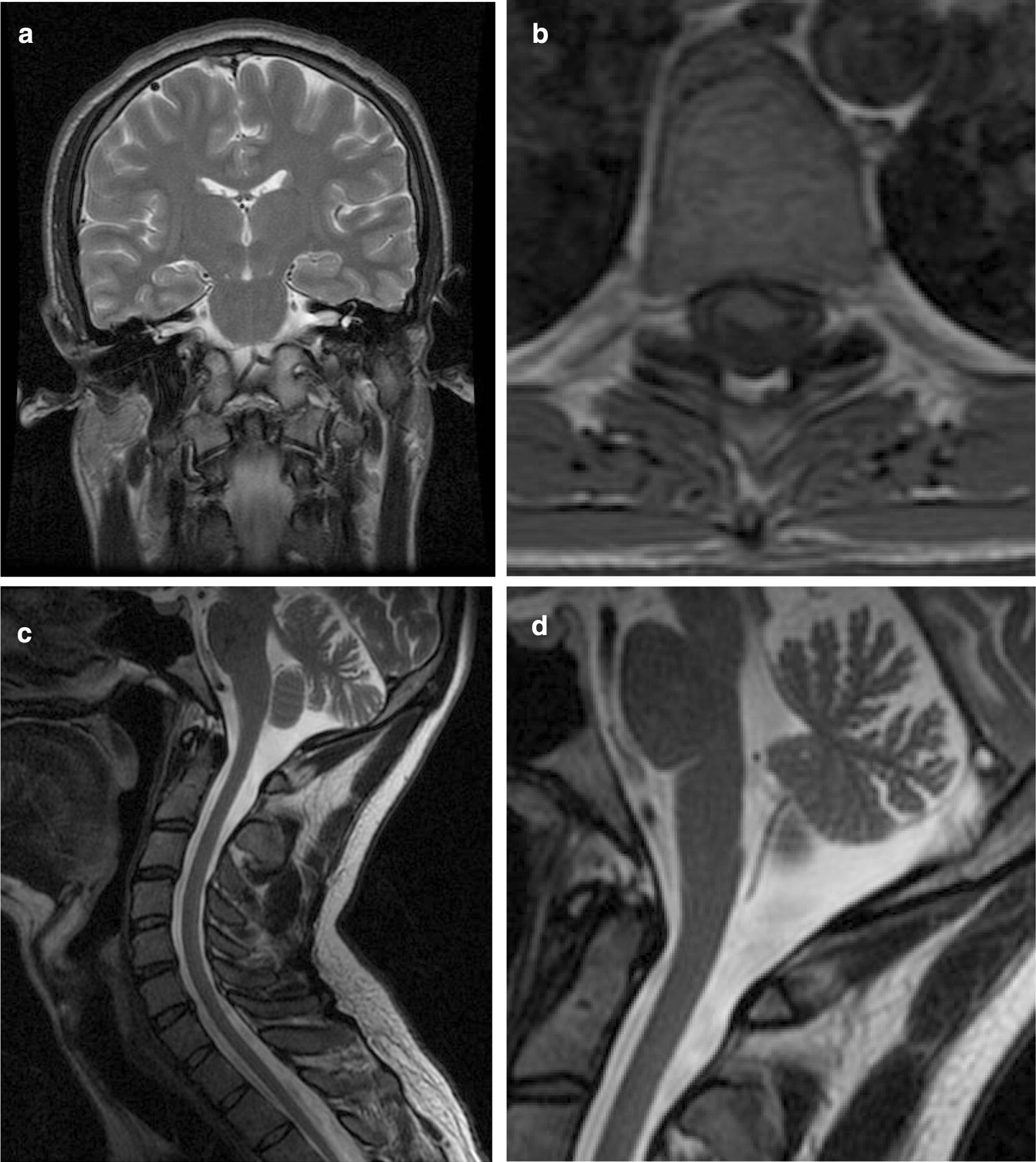
MRI images from subject II:4. **a** T2-weighted coronal slide showing normal brain parenchyma including brain stem, the inner ears and the vestibulocochlear nerves. **b** T1-weighted transversal slide of the dorsal spine where flattening of the spinal cord can be seen. **c** T2-weighted sagittal slide showing cervical kyphosis as well as cerebellar and cervical atrophy. **d** Detail of the sagittal slide to show the cerebellar atrophy more clearly

Subject II:4. He was diagnosed with sensorineural hearing loss at age 4 years. Progressive ataxia manifested at about age 20. He suffered a syncope at age 29, and was examined by a neurologist for the first time. CT scan and video-EEG were normal. On examination we found vertical and horizontal nystagmus, universal areflexia and loss of arthrokinetic sensitivity in the feet, which were cavus. Gait was unstable, and he was unable to perform tandem gait. Romberg test is positive. Endocrinologic blood tests showed autoimmune hypothyroidism and dysfunction of the gonadal axis. MRI showed cervical kyphosis, flattening of the spinal cord and cerebellar atrophy (Fig. 2). Neurophysiologic studies confirmed a profound sensorineural hearing impairment and found a dysfunction of somatosensory pathway in the posterior spinal cord and bulbo-cortical lemniscus. Electroneurography showed mixed sensitive and motor demyelinating and axonal polyneuropathy, including large and small myelinic fibers.

The patients’ mother (I:2) had been diagnosed with Parkinson’s disease, and the father (I:1) had cavus feet. Family history did not show any other relative with similar traits.

The genetic study was requested in parallel to the Service of Genetics of Hospital Universitario Ramón y Cajal (HURyC), because of the hearing impairment, and to the Functional and Translational Neurogenetics Unit (Health Sciences Research Institute Germans Trias i Pujol) (IGTP), because of the neurological signs that were observed.

In the HURyC, the clinical entity observed in this family was classified as a syndromic hearing impairment. The association of clinical signs in the three affected siblings was compatible with a diagnosis of autosomal recessive Perrault syndrome with neurological manifestations. Accordingly, all five subjects in the pedigree were genotyped for 20 microsatellite markers that map closely to the six genes known to be involved in PRLTS (Table 2). Haplotype analysis excluded linkage to *HSD17B4*, *HARS2*, *CLPP*, *LARS2* and *ERAL1*, but showed that the three siblings were haploidentical for markers close to *TWNK* (Fig. 1a). Sanger DNA sequencing of all exons and exon–intron boundaries of the *TWNK* gene revealed two potentially pathogenic sequence variants, c.85C>T (p.Arg29*) and c.1886C>T (p.Ser629Phe) (Fig. 1b), which were shared by the three siblings. Segregation analysis confirmed that the two mutations were in *trans*, the father carrying c.85C>T and the mother carrying c.1886C>T (Fig. 1a).

In the IGTP, the genetic cause of the polyneuropathy that was observed in the affected siblings was investigated by massively-parallel DNA sequencing. WES of subject II:3, with a ≥ 20× average coverage of 88.7% of the target regions, identified 72,511 variants within 1396 candidate genes under a recessive model of inheritance. Upon excluding those DNA variants with a minor allele frequency ≥ 1% in known databases, variants were prioritized by their predicted deleterious effect, amino acid conservation, and associated disease heritability. Six of these candidate variants were located in genes related to ataxia phenotype including *ATM*, *GALC*, *SPTBN2*, *SYNE1*, and *TWNK* (Additional file 1: Table S1). The best candidate variants were identified within the *TWNK* gene, c.1886C>T (p.Ser629Phe), a missense mutation that is predicted as pathogenic by all bioinformatic algorithms (SIFT score = 0.04, deleterious; PolyPhen-2 score = 0.96, damaging; MutationTaster score = 1, disease-causing) [28–30], and the nonsense mutation c.85C>T (p.Arg29*). Sanger sequencing confirmed the two sequence variants and showed the expected pattern of segregation in the family under a recessive mode of inheritance (Fig. 1a).

Mutation p.Arg29* is expected to result in a very small truncated protein lacking both domains of Twinkle, primase-related and helicase, or in no protein at all because of mRNA degradation through nonsense-mediate decay. Mutation p.Ser629Phe has not been reported previously in any database, it affects a highly-conserved residue (Fig. 1c), and it lies at the C-terminal part of the helicase domain, in a region where other mutations causing PRLTS have been reported [8, 13].

## Discussion

Most patients with rare diseases face diagnostic delays, because of the difficulties in noticing the association of clinical signs that define their particular disease [31]. In Perrault syndrome, diagnosis is further complicated by the variable expressivity of its clinical features, which is related to the age and sex of the patient, and to the diversity of genes that are involved. This is well illustrated by the familial case that we report here. Hearing loss was the first manifestation of the disease. It started in childhood (ages 3–5 years in the different patients) and it progressed quickly to become profound during the second decade of life. At that moment, the patients received a diagnosis of non-syndromic hearing impairment. The second clinical sign was the early cessation of menses in the two affected females, which took place at ages 20 and 32 years, respectively. However, the possible association of hearing loss and early amenorrhea was not noticed at that time. Neurological signs manifested later (ages 35 and 43 in the two women, respectively, and age 20 years in the male). Subsequently, genetic studies were requested, and only when the molecular results were obtained, the diagnosis of Perrault syndrome with neurological signs could be established. The same situation is observed in other patients with PRLTS [2]. Neurological signs can remain absent for years or never manifest. Male patients without neurological signs and without affected female relatives will be diagnosed of non-syndromic hearing loss, with the subsequent risk of having descendants with the syndrome. The association of hearing loss, ataxia and polyneuropathy that is found in some male patients is also observed in a large list of other neurological diseases [32]. On the other hand, the association of clinical signs could be just coincidental and not etiologically related [33].

Genetic testing can contribute to clarify the diagnosis, but it is also complicated by the genetic heterogeneity of PRLTS [34]. In spite of the identification of six genes whose mutations cause PRLTS, genetic testing for these genes does not elucidate about 60% of the clinically-diagnosed cases [2]. Although some of the cases might be just coincidental associations of signs and not true PRLTS [33], it seems clear that other genes remain to be identified. Moreover, establishing genotype–phenotype correlations with the existing data is still hampered by the small number of cases carrying mutations in each gene (Table 4). Neurological signs have been reported in patients with PRLTS and mutations in the *HSD17B4*, *TWNK*, *CLPP* and *LARS2* genes [4, 7, 8, 10–16, 20; and this work]. For *CLPP* and *LARS2*, there are also reports of patients without neurological signs [6, 7, 12, 14, 17–19], like all the patients with mutations in *HARS2* or *ERAL1* who have been reported to date [5, 9, 14]. However, it would be premature to define genotype–phenotype correlations on these bases, as the picture could change after careful follow-up of the already reported patients over the years, and after novel reports of cases with mutations in *HARS2* or *ERAL1*, still underrepresented. Moreover, different mutations in some of the six PRLTS genes may result in other diseases. For example, mutations in *LARS2* have been reported to cause hydrops, lactic acidosis, sideroblastic anemia, and multisystem failure in a female newborn [35]. Also, different mutations in *TWNK* have been reported in autosomal dominant progressive external ophthalmoplegia (AdPEO, MIM 609286) and in three autosomal recessive disorders.

**Table 4:** Overall identified mutations causing Perrault syndrome

Gene (accession number)	Mutation		References
	DNA level	Protein level	
*HSD17B4* (NM_000414.3)	c.46G>A	p.Gly16Ser	[12]
	c.244G>T	p.Val82Phe	[12]
	c.298G>T	p.Ala100Ser	[11]
	c.587C>T	p.Ala196Val	[10]
	c.650A>G	p.Tyr217Cys	[4]
	c.1704T>A	p.Tyr568*	[4]
	12-kb deletion (exons 10–13)		[10]
*HARS2* (NM_012208.3)	c.598C>G	p.Leu200Val	[5]
	c.1010A>G	p.Tyr337Cys	[14]
	c.1102G>T	p.Val368Leu	[5]
*CLPP* (NM_006012.2)	c.21delA	p.Ala10Profs*117	[16]
	c.270+4A>G		[7]
	c.425C>T	p.Pro142Leu	[16]
	c.430T>C	p.Cys144Arg	[12]
	c.433A>C	p.Thr145Pro	[7]
	c.439T>A	p.Cys147Ser	[14]
	c.440G>C	p.Cys147Ser	[7]
	c.484G>A	p.Gly162Ser	[16]
	c.624C>G	p.Ile208Met	[17]
	c.685T>G	p.Tyr229Asp	[15]
	Deletion of several exons		[16]
*LARS2* (NM_015340.3)	c.351G>C	p.Met117Ile	[12]
	c.880G>A	p.Glu294Lys	[20]
	c.899C>T	p.Thr300Met	[18]
	c.1077delT	p.Ile360Phefs*15	[6]
	c.1358G>A	p.Arg453Gln	[14]
	c.1556C>T	p.Thr519Met	[20]
	c.1565C>A	p.Thr522Asn	[6, 12, 19]
	c.1886C>T	p.Thr629Met	[6, 14]
	c.1912G>A	p.Glu638Lys	[18]
*TWNK* (NM_021830.4)	c.85C>T	p.Arg29*	This work
	c.793C>T	p.Arg265Cys	[14]
	c.968G>A	p.Arg323Gln	[12]
	c.1172G>A	p.Arg391His	[8]
	c.1196A>G	p.Asn399Ser	[12, 13]
	c.1321T>G	p.Trp441Gly	[8]
	c.1519G>A	p.Val507Ile	[8]
	c.1754A>G	p.Asn585Ser	[8]
	c.1802G>A	p.Arg601Gln	[13]
	c.1886C>T	p.Ser629Phe	This work
*ERAL1* (NM_005702.3)	c.707A>T	p.Asn236Ile	[9]

The *TWNK* gene codes for the 684-aa monomer of the mitochondrial DNA helicase twinkle, which localizes to the mitochondrial matrix and is thought to play a key role in mtDNA replication [36, 37]. The enzyme is a ring-shaped homohexamer, each monomer containing a primase-related domain (residues 79–346), a helicase domain (residues 384–635) and a linker region between these two domains [38]. The linker region is critical for hexamerization and helicase activity [39]. Most mutations causing AdPEO are missense and affect residues either within the linker or in close proximity to it in the tertiary structure of the protein [40, 41]. They lead to either destabilization of the monomers (flexibility of the linker region is diminished) or to defective hexamerization (inhibition of the ring closure or changing the number of subunits within the ring), which in turn impair the helicase activity and cause replication stalling [40, 41]. On the other hand, recessive mutations include a few truncating variants, including deletions causing frameshifts, nonsense and splice-site mutations (to our knowledge, p.Arg29* is the first reported truncating mutation in a patient with PRLTS). However, the majority of recessive mutations are missense, some of them affecting residues in the primase-related domain, but most of them affecting the helicase domain (like p.Ser629Phe in our patients) [42]. They result in three different phenotypes: Perrault syndrome type 5 (MIM 606075) [8], infantile-onset spinocerebellar ataxia (IOSCA) [43] and hepatocerebral syndrome [44], although the last two disorders are now grouped under a unique code (MIM 271245). In fact, the three diseases constitute a phenotypic continuum ranging from the relatively milder form (PRLTS5) to the most severe form (hepatocerebral syndrome, with acute liver failure). Hearing impairment is reported in patients of the three conditions, and ovarian insufficiency may be also a shared feature if not masked by a premature lethality. Therefore, classification of cases depends on the age of onset and severity of other clinical signs. In IOSCA and hepatocerebral syndrome, the neurological signs manifest in early childhood [42–44], whereas in PRLTS5 the onset takes place much later in life [8, 12–14], as also shown in patients of family SH19. In hepatocerebral syndrome, the liver is severely affected [44]. Of note, subject II:3 of family SH19 shows an increase of liver enzymes of unknown cause. The existence of this phenotypic continuum is also supported by the spectrum of mutations in the *TWNK* gene. Whereas there is no overlap between the mutations causing dominant or recessive phenotypes, this overlap does exist among the recessive diseases [42]. For example, mutation p.Tyr508Cys has been reported in cases of IOSCA (it is a founder mutation in Finland) [43], but also in hepatocerebral syndrome [44, 45]. Moreover, mutation p.Arg29* has been reported in a case of hepatocerebral syndrome [45] and in PRLTS5 (this work). It is remarkable that a child with hepatocerebral syndrome is compound heterozygous for p.Arg29* (also seen in PRLTS5) (this work) and p.Tyr508Cys (frequent in IOSCA) [45]. It is clear that clinical and genetic characterization of additional patients, like those reported here, is needed to progress in understanding the genotype–phenotype correlations in subjects with mutations in *TWNK*.

## Conclusions

Genetic testing is a useful tool to confirm a tentative clinical diagnosis of Perrault syndrome, although the genetic cause remains unidentified in over half of the cases. Studies on genotype–phenotype correlation from the hitherto reported cases indicate that all patients with Perrault syndrome caused by *TWNK* mutations will manifest neurological signs in adulthood. Recessive disorders caused by *TWNK* mutations constitute a phenotypic continuum, so that establishing genotype–phenotype correlations is a challenging task that still needs identification and molecular and clinical characterization of novel cases.

## Supplementary information

**Additional file 1: Table S1.** Relevant mutations obtained by whole-exome sequencing of subject II: 3.

## Data Availability

The datasets used and/or analysed during the current study are available from the corresponding author on reasonable request. However, the Whole Exome Sequencing datasets generated and/or analysed during the current study are not publicly available because of the European Union General Data Protection Regulation (GDPR).

## Abbreviations

Aa: amino acid
AdPEO: autosomal dominant progressive external ophthalmoplegia
CT: computed tomography
EEG: electroencephalography
HURyC: Hospital Universitario Ramón y Cajal
IGTP: Institute Germans Trias i Pujol
IOSCA: Infantile-Onset SpinoCerebellar Ataxia
MRI: Magnetic Resonance Imaging
mtDNA: mitochondrial DNA
PRLTS: Perrault syndrome
WES: whole exome sequencing
