# Supplementary material for: Perrault syndrome with neurological features in a compound heterozygote for two TWNK mutations: overlap of TWNK-related recessive disorders
Source: J Transl Med. 2019 Aug 28;17:290. doi: 10.1186/s12967-019-2041-x (PMC6712801; doi:10.1186/s12967-019-2041-x)
Supplement: Supplementary file 1 — Additional file 1: Table S1. Relevant mutations obtained by whole-exome sequencing of subject II: 3. [file 12967_2019_2041_MOESM1_ESM.docx]

**Table S1.** Relevant mutations obtained by whole-exome sequencing of subject II: 3

| **GENE** | **CHROMOSOME POSITION (GRCh37 - hg19)** | **ZYGOSITY** | **NUCLEOTIDE VARIATION ^a^** | **PROTEIN CHANGE** | **VARIANT EFFECT** | **EXISTING VARIATION** | **PATHOGENICITY PREDICTION ^b^** |
| --- | --- | --- | --- | --- | --- | --- | --- |
| ***TWNK*** | **chr10:102748052** | **HET.** | **c.85C>T** | **p.Arg29Ter** | **stop gained** | **rs1292672301** | **-** |
|  | **chr10:102753098** | **HET.** | **c.1886C>T** | **p.Ser629Phe** | **missense variant** | **-** | **DELETERIOUS** |
| *ATM* | chr11:108183167 | HOMO. | c.5948A>G | p.Asn1983Ser | missense variant | rs659243 | NEUTRAL |
| *GALC* | chr14:88417095 | HOMO. | c.1093-4delT | - | splice region variant | rs11300320 | N.S.A. |
| *SPTBN2* | chr11:66472274 | HOMO. | c.2473A>G | p.Ser825Gly | missense variant | rs4930388 | NEUTRAL |
| *SYNE1* | chr6:152540278 | HOMO. | c.5476T>G | p.Phe1826Val | missense variant | rs2147377 | NEUTRAL |

^a^ Nucleotide variations were annotated regarding to the following transcripts: C10orf2: ENST00000311916.2; ATM: ENST00000278616.4;

GALC: ENST00000393568.4; SPTBN2: ENST00000533211.1; SYNE1: ENST00000356820.4.

^b^ Condel, Sift and Polyphen were used for pathogenicity prediction of missense variants. Human splicing finder 3.1 was used for splicing effect prediction.

Het, Heterozygous; Homo, Homozygous; N.S.A, Non-splicing alteration.
